# Evaluating outcomes of psychological support services in minimum security prisons in England: a quasi-experimental retrospective cohort study using propensity score matching and survival analysis

**DOI:** 10.1136/bmjopen-2025-110540

**Published:** 2026-03-30

**Authors:** Georgina Mathlin, Hannah Jones, Carine Lewis, Claudia Cooper, Mark Freestone

**Affiliations:** 1 Centre for Psychiatry and Mental Health, Wolfson Institute of Population Health, Queen Mary University of London, London, UK; 2 His Majesty’s Prison and Probation Service, London, UK; 3 The Tavistock and Portman NHS Foundation Trust, London, UK

**Keywords:** *Offender Personality Disorder*, open prisons, minimum security prisons, propensity score matching, survival analysis

## Abstract

**Abstract:**

**Objectives:**

To test whether individuals identified as having a likely personality disorder and high reoffending risk, who opted in to psychologically informed support in minimum security prisons (Pathways Enhanced Resettlement Service (PERS)), were (a) less likely to return to more restrictive closed prison conditions, and (b) released to the community sooner, compared with matched non-PERS users.

**Design:**

Quasi-experimental retrospective cohort design using routine PERS and UK Ministry of Justice administrative data, for all clients enrolled on PERS between 1 April 2019 and 30 April 2022.

**Setting:**

Five minimum security male prisons in England.

**Participants:**

258 PERS users matched for ethnicity, age at first offence and sentencing, education, sentence type, personality disorder screening, risk of reoffending scores and historical drug and alcohol use to 358 controls.

**Intervention:**

PERS.

**Results:**

PERS users waited longer for community release (HR=0.16, 95% CI 0.03 to 0.71) and had a lower likelihood of return to closed conditions prisons (HR*=*0.73*,* 95% CI 0.53 to 0.99) than matched controls.

**Conclusions:**

PERS supports individuals to remain in open conditions for longer; however, further evaluation is needed to understand why PERS users waited longer for release, and if this was associated with a more successful release, without return to prison or reoffence.

**STRENGTHS AND LIMITATIONS OF THIS STUDY**

- All individuals who engaged with the English national Pathways Enhanced Resettlement Service (PERS) within the study period are included in the analysis, enabling population-level inferences to be made.
- We were unable to control for several confounders that may have influenced the outcome, including previous failure in a minimum security prison, time in the open prison before joining PERS, time remaining on a sentence and psychological well-being.
- Just under a quarter of outcome data across the covariates was missing; use of multiple imputation can only partially mitigate this limitation.

## Introduction

 An estimated two-thirds of the prison population meet personality disorder criteria.^1 2^ Personality disorder is a complex mental health condition associated with difficulties with interpersonal relationships, identity and behaviours which deviate from cultural expectations.^3^ Individuals with personality disorder have poor health and psychiatric outcomes, increased risks of violence^4^,^7^ and reoffending.^8^ Although it is possible to improve psychological well-being and reduce offending behaviour in this population,^9^ therapeutic engagement is challenging, with high levels of treatment drop-out.^10^

Minimum security prisons are less structured prison environments with less control and restriction of individuals,^11^ which can be difficult to navigate for people with a likely diagnosis of personality disorder.^12 13^ Minimum security prisons seek to aid community resettlement, providing opportunities for offenders to reintegrate with communities, building links with family, housing services, volunteering and identifying employment opportunities.^14^ Retaining a position in a minimum-security prison can be difficult, due to low thresholds for being returned to a higher security establishment, including substance misuse lapses, feeling threatened or bullied and being violent, issues which are especially prevalent for people with a likely diagnosis of personality disorder.^15 16^ This perceived insecurity can lead to reluctance to seek mental health support.^17^ Post-release recidivism and mortality is lower for individuals released from minimum, relative to higher security prisons.^11 18^ However, there is little research about how minimum security prisons might best support offenders to realise resettlement goals,^19 20^ especially for individuals with a likely diagnosis of personality disorder.

The Offender Personality Disorder Pathway (OPDP) provides psychologically informed services across the UK criminal justice system for people with a likely diagnosis of personality disorder.^17^ It aims to reduce repeat serious offending and improve psychological well-being.^21^ In 2019, five Pathways Enhanced Resettlement Services (PERS) opened in English male minimum security prisons. UK minimum security prisons, known as open prisons, house adult males with low risks of absconding and/or risk to the public due to the nature of their offence, and individuals near the end of a long sentence who are stepped down security categories to prepare for release.^19^ PERS aim to support transitions by supporting moves from closed to open prison, reducing the likelihood of individuals being returned to closed prisons and increasing the likelihood of successful moves from open prison to the community for people with a likely diagnosis of personality disorder. All new open prison arrivals with indeterminate sentences or deemed at high risk of self-harm are screened against standard OPD criteria by PERS staff^17^; those in the ‘high-risk’ category of reoffending; who have been at risk of self-harm or suicide in the last 12 months or who have previously been returned from open prison or the community to closed conditions are invited by a staff member to join PERS, which is voluntary.

PERS comprises three core components. First, a risk formulation for each individual is developed to understand and manage their potential difficulties transitioning into open prison and re-entering the community. Key work sessions then provide a space for PERS staff to work alongside service users to identify support and opportunities to assist release planning and form community links. All service users have access to a drop-in service for informal, responsive support. PERS relies on a relational model and through the range of support on offer aims to increase attachment security of individuals with a likely diagnosis of personality disorder. On average, people engage with PERS for 7 months; however, engagement can last up to 2.5 years. The amount of engagement with PERS may fluctuate and is responsive to the needs of the individual, for example, more key work sessions and increased use of the drop-in support may occur at times of stress, such as in the lead up to parole hearings.

Two qualitative studies exploring the experiences of PERS service users and staff^22 23^ have highlighted the value of support provided around difficult transitions into open prison, the development of trusting relationships and benefit of individualised support. No further studies have been conducted to evaluate PERS, and this study is the first to empirically explore whether PERS use is associated with supporting transitions.

Ensuring that offenders are offered evidence-based, effective psychological support to enable transitions has major potential benefits for offenders, staff and society. To explore whether the relational model of PERS, which aims to support moves from close to open prison and open prison into the community, is meeting its intended outcomes, we hypothesised that PERS users within five English male open prisons would return slower to closed conditions and be released into the community quicker, compared with a matched group of non-PERS users.

## Methods

### Study design and data sources

We carried out a quasi-experimental retrospective cohort study, using secondary data only, comparing time to return to closed prison and time to community release of PERS users to a matched control group of non-PERS users, in non-identifiable administrative data collected by PERS and central Ministry of Justice (MoJ) databases: NDelius, OASys and NOMIS are databases held by the MoJ where routine service data is entered for all offenders in the UK prison and probation system by prison and probation officers. We used the PERS dataset (service-level data) to identify all PERS users between 01.04.2019 (the date the five services formally opened) until 30 April 2022 (N=269); we linked PERS users to NDelius data across the same period (n=38 624) a national probation browser-based interface for case management which contains demographic information about offenders and OASys data, which contains information about the likelihood of reoffending, offending-related needs, risk of harm to the individual and others and supervision plans.^24^ The likelihood of reoffending is calculated through a statistical algorithm. The identification of non-PERS users happened at this stage. Cases obtained through the matching were then linked to NOMIS (National Offender Management Information System), an operational database containing information about movements in and out of prisons to identify first which matched non-PERS users were in open prisons during the study period and second the outcomes of interest (return to closed conditions, release into the community or remaining in the open prison). A final research dataset including information on service use (PERS or non-PERS) and outcomes (returned to closed conditions, release to the community or remaining in open prison) was used to conduct the analysis.

The datasets were linked using the offender’s Date of Birth and Case Reference Number, a unique identifier assigned to each prison case, attached to the individual, rather than the prison episode.

### Study setting participants

All adult males who voluntarily joined PERS from 01.04.2019 (the data first service opened) until 30 April 2022 were included in the ‘PERS users’ group (N=269). This represents complete coverage of the population and is representative of all service users during this period. PERS is delivered across five male open prisons across England. Two of the open prisons hold offenders with sexual offences.

A control group of ‘non-PERS users’ (adult males entering any open prison between 1 April 2019 and 30 April 2022 who had never used PERS, including those who were approached but declined) was identified using propensity score matching (PSM) from the NDelius dataset, with no individuals excluded from this dataset prior to the PSM. The MatchIt^25^ and optmatch^26^ R packages were used. Optimal matching was chosen as it minimises the average absolute distance across all matched pairs. We selected covariates for the PSM to align with PERS eligibility criteria and baseline characteristics. Baseline characteristics included ethnicity (Asian, Black, mixed, other and white), age at sentencing and first offence, education (any qualification or no qualifications), history of drug use (no drugs ever misused, or drugs misused) and history of alcohol use (no problems, some problems or significant problems). PERS eligibility criteria matched on included sentence type (determinate and indeterminate, including life sentences), OASys PD screen (individuals with 7+ screening items or <7 items but had another criterion: childhood difficulties, history of mental health difficulties, problematic behaviour, self-harm/ suicide attempts) were identified as meeting the OPDP criteria,^27^ history of self-harm (yes, no) and Offender Group Re-conviction Scale score (version 3; OGRS3, Howard *et al*, 2009),^28^ a predictor of re-offending within 2 years of release. Matching was conducted at the episode level and therefore an individual who used PERS more than once in the study period was matched based on their covariates at the time of each episode.

Before matching the study participants, we visualised patterns of missing and non-missing data across the dataset of baseline demographics and covariates for PERS and unmatched non-PERS cases. 24.6% of data across the co-variables were missing. We implemented a function to compare missing and non-missing values on each covariate and found a significant difference across each covariate, suggesting the missing data was dependent on another observed variable in the dataset and was subsequently treated as missing at random.^29^ Missing Imputation Chained Equations were used with the R package mice^30^ to impute missing data (m=5) for all variables with missingness apart from ethnicity (n=11), as this is a characteristic not considered suitable for imputation. Logistic regressions were used to impute the binary variables (education, drugs ever misused), polytomous logistic regressions for the categorical variables (alcohol misuse in the past) and predictive mean matching for the continuous variables (age at first conviction, OGRS score). The imputation was checked, and all imputed data followed the same distribution as the original data.

A four to one ratio of matching was used, identifying four non-PERS service users for each PERS service user. The matching took place prior to individuals being identified as having been in an open prison (when the data was linked to NOMIS) and therefore the final number of controls to service user ratio was less than four to one. The 4:1 ratio was therefore chosen to ensure that the control group still included a sufficient sample in the Cox Proportional hazard models. The PSM model, matching details and balance plots are presented in online supplemental table S1 and figures S1-S2, alongside an alternatively considered matching approach of nearest neighbour (online supplemental table S2 and figures S3-S4).

To assess the robustness of the findings to the matching process, a sensitivity analysis was conducted in which PSM was performed after restricting both groups to individuals confirmed as having been in an open prison during the study period (April 2019 to April 2022). A 1:1 matching ratio was used in the sensitivity analysis with the same optimal matching algorithm and covariates were used as in the primary analysis. Results of the sensitivity analysis are presented in (online supplemental table S3).

### Outcomes

Our primary outcome was time (in days) from start of the index open prison stay, to (a) first return to a closed condition prison and (b) time to release into the community. Outcomes were censored at the date of event, or 30 April 22.

### Statistical analysis

Descriptive statistics of demographic, psychosocial and offending characteristics of those returned to closed conditions, released into the community and remaining in the open prison are presented. Differences between the groups were explored using χ² tests of independence for categorical variables and one-way analysis of variance tests for continuous variables. Kaplan-Meier curves were generated plotting PERS user against non-PERS unadjusted time-to-event patterns for release to the community and return to closed conditions using the survminer R package.^31^ We explored whether the likelihood of being returned to closed conditions prison and released into the community differed between PERS users and the matched group of offenders who had not used PERS. Cox Proportional Hazard models were estimated using partial likelihood with the survminer R package.^31^ The models were clustered by the individual using cluster-robust SEs to account for people who were in open prison more than once throughout the study period (N=69; n=29 (PERS), n=40 (non-PERS), M=2). A univariable (service use only) and adjusted (controlling for all covariates included in the PSM to account for residual confounding) model is reported. The initial models (N=638) violated the proportional hazard test (see online supplemental tables S4-S7), indicating time-varying effect of PERS service use, ethnicity and age at first conviction on return to closed conditions and release into the community. The model exploring the effect of PERS on risk of return to closed conditions was re-run only including the first 500 days after entering open prison. For the univariable model, the proportional hazard assumption was no longer violated, and this model is reported (N=507). For the adjusted model, ethnicity still violated the proportional hazard assumption. The truncated model for only the first 500 days was re-run, stratifying by ethnicity, and the proportional hazard test was not violated; therefore, this adjusted model is reported (N=507). The model investigating the effect of PERS on release into the community was re-run looking at 150 days after entering the open prison. The proportional hazard assumption was no longer violated and therefore these models are reported.

### Role of the funding source

The funder of the study had no role in study design, data collection, data analysis, data interpretation, or writing of the report.

### Patient and public involvement

An ex-PERS service user who had been released into the community was involved in the design of this study as they had expressed an interest in being involved in research to their key worker. The ex-service user provided feedback on the broader evaluation plan of PERS, including this study, and agreed that exploring return to closed conditions and release to the community outcomes was an important first step in understanding the impact of PERS as these are what PERS service users focus on during their time in open prison (avoiding returns and progressing towards release).

## Results

Between April 2019 and April 2022, 269 individuals were recorded as PERS users. 11 people had missing ethnicity data and were removed from the analysis, leaving 258 individuals included in the PERS user group. 258 individuals were matched to a control group of 1076 matched controls who had not used PERS. Out of the 1076 controls identified through the matching, only 358 had been in an open prison between April 2019 and April 2022, so this was the final sample size of the matched control group. Balance measures for the prematched and postmatched sample are presented in online supplemental table S8.

Table 1 provides descriptive statistics of the three outcomes: remaining in open prison (n=239), returned to closed conditions (n=237) and release to the community (n=162). Due to the PSM, the groups are similar; however, individuals released to the community were significantly more likely to be on a determinant sentence compared with individuals returned to closed conditions and remaining in open prison, who had a higher proportion of being on indeterminate sentences. People returned to closed conditions were younger and younger at first conviction than people remaining in the open prison or released to the community. People returned to closed conditions prisons also had significantly higher average OGRS3 scores than those remaining in the open prison and released into the community.

**Table 1:** Descriptive statistics of returned to closed, released to community and currently in open prison subgroups

	Open prison	Return to closed	Release to community	P value
Ethnicity (n, %)				
White	172 (71.97%)	166 (70.04%)	111 (68.52%)	
Black	47 (19.67%)	45 (19.00%)	30 (18.52%)	
Mixed	7 (2.93%)	13 (5.49%)	4 (2.47%)	
Refusal	11 (4.60%)	7 (2.95%)	8 (4.94%)	
Asian	2 (0.84%)	6 (2.53%)	9 (5.56%)	
Education (n, %)				
Any qualification	201 (84.10%)	202 (85.23%)	139 (85.80%)	
No qualification	38 (15.90%)	35 (14.77%)	23 (14.20%)	
Sentence type (n, %)				
Determinant	67 (28.03%)	69 (29.90%)	100 (61.73%)	
Indeterminant	157 (65.69%)	155 (65.40%)	59 (36.42%)	
Young offender	15 (6.28%)	13 (5.49%)	3 (1.85%)	
OPD screen (n, %)				
Screened in	47 (19.67%)	63 (26.58%)	27 (16.67%)	
Not on pathway	192 (80.33%)	174 (73.41%)	135 (83.33%)	
Drugs ever misused (n, %)				
No	12 (5.02%)	4 (1.69%)	7 (4.32%)	
Yes	227 (94.98%)	233 (98.31%)	155 (95.68%)	
Alcohol misuse (n, %)				
No problems	87 (36.40%)	82 (34.60%)	63 (38.89%)	
Some problems	54 (22.59%)	48 (20.25%)	45 (27.78%)	
Significant problems	98 (41.00%)	107 (45.15%)	54 (33.33%)	
History of self-harm (n, %)				
Yes	164 (68.62%)	165 (69.62%)	99 (61.11%)	
No	75 (31.38)	72 (30.38%)	63 (38.89%)	
Age (M, SD)	30.69 (9.02)	27.94 (7.45)	32.56 (10.92)	<0.001
Age first conviction (M, SD)	17.23 (5.12)	15.60 (3.87)	17.93 (6.78)	<0.001
OGRS3 (M, SD)	49.94 (22.70)	55.89 (20.05)	49.70 (22.28)	0.003

Note: Individuals given a Young Offender sentence type were 18 or over and, in an adult, only open prison during the study period.

OGRS3, Offender Group Re-conviction Scale version 3; OPD, offender personality disorder.

### Time to release to the community

**Figure 1:**
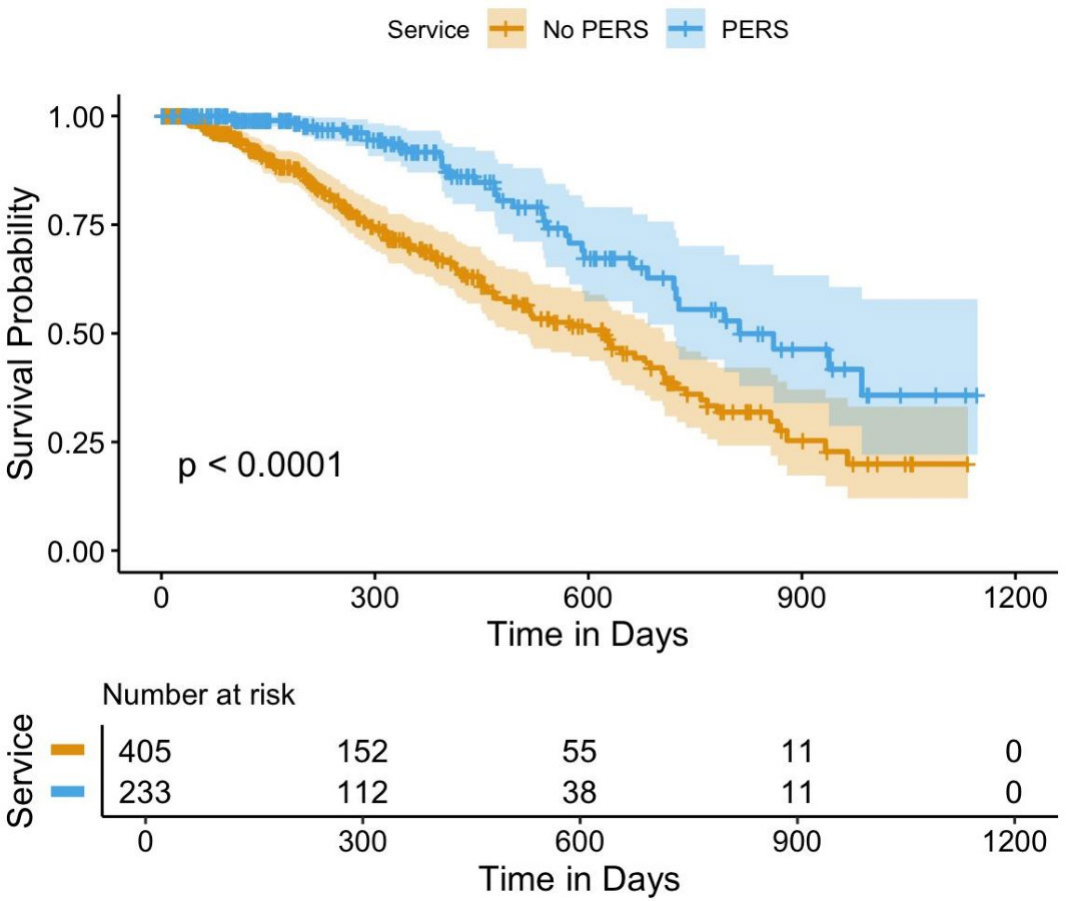
Kaplan-Meier curve PERS versus non-PERS release into the community. PERS, Pathways Enhanced Resettlement Service.

The mean time spent in an open prison before being released into the community was 375 days (median=318 days; IQR=194–532). Across the study period, 162 people were released into the community. The 239 people remaining in open prison and 237 people returned to closed conditions were included as censored observations in the models exploring time to release to the community. The Kaplan-Meier curve shows the probability of being released into the community over 1200 days separated by service use, which reflects the direction of the HR estimates reported in the Cox Proportional Hazards models of non-PERS users being released to the community quicker than PERS users (figure 1).

407 people (PERS: n=246, non-PERS: n=161) were included in the release to the community analysis (analysing data only after individuals had been in the open prison for more than 150 days). The mean time to release to the community for the non-PERS group was 422 days, compared with 454 days in the PERS group. PERS service users were 6.25 times less likely to be released to the community compared with the non-PERS sample (p=0.016, HR=0.16, 95% CI 0.03 to 0.71) (table 2).

**Table 2:** Cox proportional hazard unadjusted and adjusted models

	Univariable			Adjusted*		
	HR	95% CI	P value	HR	95% CI	P value
Return to closed conditions						
Service use PERS (reference)	0.82	0.61 to 1.09	0.164	0.73	0.53 to 0.99	0.041
Release to the community						
Service use PERS (reference)	0.10	0.02 to 0.42	0.001	0.16	0.03 to 0.71	0.016

* Adjusted for age, education (no qualification/qualification), sentence type (indeterminate, determinate, young offender), OPD screen (yes/no), age at first conviction, drugs ever misused (yes/no), alcohol misuse (significant, some, no), history of self-harm (yes/no), ORGS3 2-year reconviction score. Stratified by ethnicity (Asian, Black, Mixed, Refused, White).

OPD, offender personality disorder; ORGS3, Offender Group Re-conviction Scale version 3; PERS, Pathways Enhanced Resettlement Service.

### Time to return to closed conditions

The mean time spent in open prison for the 237 people who were returned to closed conditions was 203 days (median=134 days; IQR=309–650 days). The 239 people still in open prison at the end of the study period (31.04.2022) and the 162 individuals released to the community were included in the models exploring time to return to closed conditions as censored observations.

**Figure 2:**
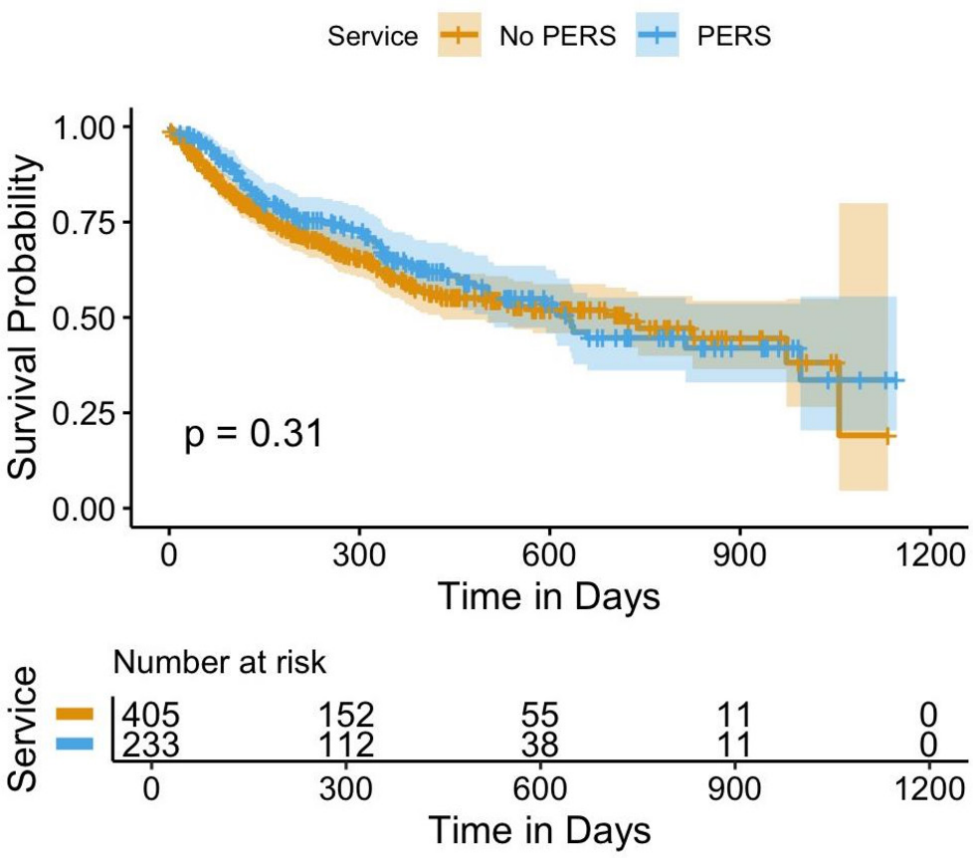
Kaplan-Meier curve for time to return to closed prison. PERS, Pathways Enhanced Resettlement Service.

The Kaplan-Meier curve (figure 2) shows the probability of being returned to closed conditions across 1200 days by service use and reflects the results of the Cox Proportional Hazard model of PERS users being returned to closed conditions at a slightly quicker rate than non-PERS until 500 days where a clear pattern is no longer established. 507 (PERS: n=329, non-PERS: n=178) people were included in the analysis of time to return to closed conditions in the first 500 days from entry into the open prison. The mean time to be returned to closed conditions for the non-PERS group was 185 days, compared with 220 days for the PERS group. PERS service use was associated with a significantly reduced likelihood of return to closed conditions (p=*0*.041, HR=0.73, 95% CI 0.53 to 0.99). The HR indicates PERS service users were 1.39 times less likely to be returned to closed conditions compared with the non-PERS sample (table 2).

## Discussion

This is the first quantitative study to explore whether a nationally provided, specialist psychological service for people in minimum security prisons supports individuals to delay returning to higher security prisons. We hypothesised that PERS users would return to closed conditions slower compared with non-PERS users; they did, returning on average a month later. We also expected PERS service users would have an increased rate of release to the community compared with non-PERS service users, but contrary to this, they were released around a month later.

There are several possible explanations for our findings. The lower likelihood of return to closed conditions among PERS users could indicate that PERS is supporting offenders to remain in open conditions by changing the behavioural or emotional outcomes for individual clients. This is supported by qualitative work reporting that following the shock of adapting to open conditions, PERS service users feel valued, and the person-centred approach of the service allows trusting, therapeutic relationships to develop which supports the progression through their sentence.^23^ Alternatively, open prisons may bring benefits at a prison level. An aim of PERS was for staff to share their psychological understanding with the wider prison workforce,^32^ and the findings may suggest staff take on board PERS approaches in their wider work and develop new offender management strategies or a higher tolerance for risky behaviours compared with standard open prisons.

Although unexpected, the finding that PERS users are released to the community and returned to closed conditions slower than non-PERS users suggests that overall individuals using PERS remain in open conditions prisons for longer. If the impact of the service is for offenders to spend longer in open prison, receiving psychological support for reintegration, and less time in closed conditions, this could have benefits to society and individuals. A longer duration in open prison may mean more time to slowly reintegrate into the community, build strong links with the community and family and there possibly lead to a greater chance of success when released. This should be tested and explored in future research.

Some limitations of this study should be considered. No data was available to match the comparison group on previous failure in a minimum security prison, time in the open prison before joining PERS, time remaining on a sentence and psychological well-being; therefore, a biased estimate of the effect of PERS on the likelihood of return to closed conditions and release to the community may have been reported. Furthermore, the matching of the control group occurred at the level of all possible individuals in custody before being able to identify which individuals in the control group had been in an open prison during the study period. This meant equal numbers were not in the treatment and control group and there were differences in the number of controls matched to the study participants which may have introduced confounding. A sensitivity analysis conducted matching only on the controls in open prison during the study period which produced directionally consistent results for the return to closed conditions outcome. However, the rarity of the release to community outcome in the sensitivity sample (four events) precluded meaningful sensitivity testing for this outcome.

The analysis relied on routinely collected administrative data which was not originally collected for data analysis. Although the wealth of data has provided useful insights into the effect of PERS, there was a substantial amount of missing data across the covariates, which meant missing imputation was used and a complete cases sensitivity analysis was not possible due to a small sample size of the PERS group when exploring the feasibility of a complete case analysis. Furthermore, a sensitivity analysis including the first entry to the open prison only was not feasible due to the final analytical dataset not including the specific date of entry to the prison (only how many days from entry until an outcome). Missing imputation allows missing data points to be included in the analysis and subsequently reduces the potential bias of losing the data. However, it was not feasible to impute some variables, such as ethnicity, meaning 11 people with missing ethnicity information were removed from the analysis, which may have biased findings. Once individuals were returned to closed conditions or released, they were censored, so we were unable to capture any subsequent moves, back to open prison or to the community which we are aware are common pathways into the community (individuals often have multiple attempts at open prison prior to a release or release from closed conditions). We compared those who opted into receiving support to a cohort who were either not offered support or declined it. It is possible that willingness to receive help was a critical confounder. It is also possible that PERS may have been commissioned in open prisons that were already more supportive and tolerant environments.

It is unclear from the current study whether individuals who use PERS and are released into the community reintegrate successfully into and remain in the community offence-free. Future research should explore the long-term impact of PERS on post-release outcomes such as housing, subsequent mental health and health service use, employment and offending outcomes such as recall and reoffending.

In conclusion, this study provides evidence that offenders with personality disorders who opted into a psychological support service spent longer in a minimum security prison; on average, they returned to closed conditions a month later, but they were also released around a month later. This study provides useful information for policymakers about how PERS influences lengths of stays in open prisons; it lengthens them and reduces the likelihood of return to closed conditions. Given the economic and potential well-being benefits of open versus closed prisons, this evidence could support further investment in PERS services. However, further research is needed to understand whether the additional time within a minimum security environment supports long-term successful reintegration in the community through a continued longitudinal assessment of PERS outcomes as the programme continues and more people are released who have used the service. Relatedly, future research should explore whether PERS reduces the likelihood of individuals with personality disorder reoffending, and whether the service is cost-effective.

## Supplementary material

10.1136/bmjopen-2025-110540 online supplemental file 1

## Data Availability

No data are available.
