# Supplementary material for: Evaluating outcomes of psychological support services in minimum security prisons in England: a quasi-experimental retrospective cohort study using propensity score matching and survival analysis
Source: BMJ Open. 2026 Mar 30;16(3):e110540. doi: 10.1136/bmjopen-2025-110540 (PMC13052529; doi:10.1136/bmjopen-2025-110540)
Supplement: online supplemental file 1 [file bmjopen-16-3-s001.docx]

Supplemental Material

Table S1. Logistic Regression Model Used to Estimate Propensity Scores

|  | estimate | std.error | statistic | p.value | OR | CI_lower | CI_upper |
| --- | --- | --- | --- | --- | --- | --- | --- |
| Intercept | -21.239 | 235.226 | -0.090 | 0.928 | 0.000 | 0.000 | 1.009810e+191 |
| Ethnicity |  |  |  |  |  |  |  |
| Asian | 12.277 | 235.226 | 0.052 | 0.958 | 214768.156 | 0.000 | 3.631678e+205 |
| Black | 13.598 | 235.226 | 0.058 | 0.954 | 804173.757 | 0.000 | 1.358994e+206 |
| Mixed | 12.897 | 235.226 | 0.055 | 0.956 | 399156.619 | 0.000 | 6.746930e+205 |
| Refusal | 14.611 | 235.226 | 0.062 | 0.950 | 2214677.319 | 0.000 | 3.743663e+206 |
| White | 13.078 | 235.226 | 0.056 | 0.956 | 478244.872 | 0.000 | 8.081625e+205 |
| Age | 0.000 | 0.006 | 0.046 | 0.964 | 1.000 | 0.988 | 1.012000e+00 |
| Education | -0.224 | 0.145 | -1.549 | 0.121 | 0.799 | 0.601 | 1.061000e+00 |
| Sentence type |  |  |  |  |  |  |  |
| Indeterminant | 1.700 | 0.119 | 14.239 | 0.000 | 5.473 | 4.331 | 6.915000e+00 |
| Young offender | 0.919 | 0.297 | 3.098 | 0.002 | 2.507 | 1.402 | 4.484000e+00 |
| OPD screen | 0.853 | 0.127 | 6.746 | 0.000 | 2.348 | 1.832 | 3.008000e+00 |
| Age first convicted | -0.021 | 0.013 | -1.642 | 0.101 | 0.979 | 0.955 | 1.004000e+00 |
| Drugs ever misused | 2.158 | 0.391 | 5.519 | 0.000 | 8.658 | 4.023 | 1.863500e+01 |
| Alcohol misuse |  |  |  |  |  |  |  |
| Some alcohol misuse problems | -0.035 | 0.145 | -0.238 | 0.812 | 0.966 | 0.966 | 1.284000e+00 |
| Significant alcohol misuse problems | 0.159 | 0.133 | 1.193 | 0.233 | 1.172 | 0.903 | 1.522000e+00 |
| History of self harm | 1.634 | 0.114 | 14.314 | 0.000 | 5.122 | 4.095 | 6.406000e+00 |
| OGRS | 0.001 | 0.003 | 0.180 | 0.857 | 1.001 | 0.995 | 1.006000e+00 |

PSM matching details:

Method: Optimal

Ratio: 4 controls per treated case

Distance: logistic regression propensity score

Replacement: no

Software: “MatchIt” and “optmatch” R packages.

PSM Balance plots

Figure S1. Histogram plots


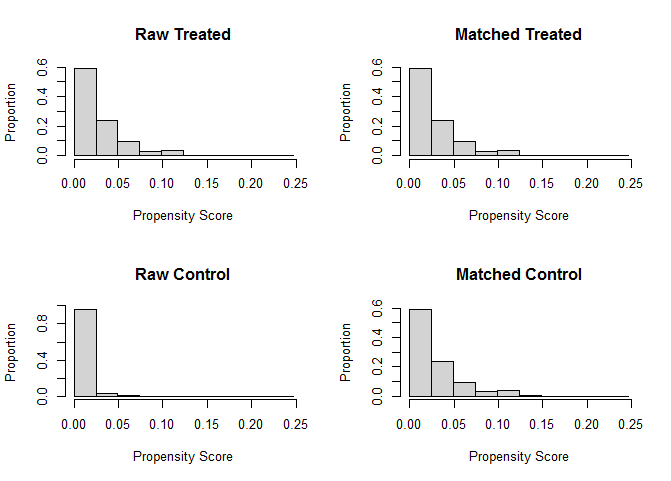


Figure S2. Jitter plot.


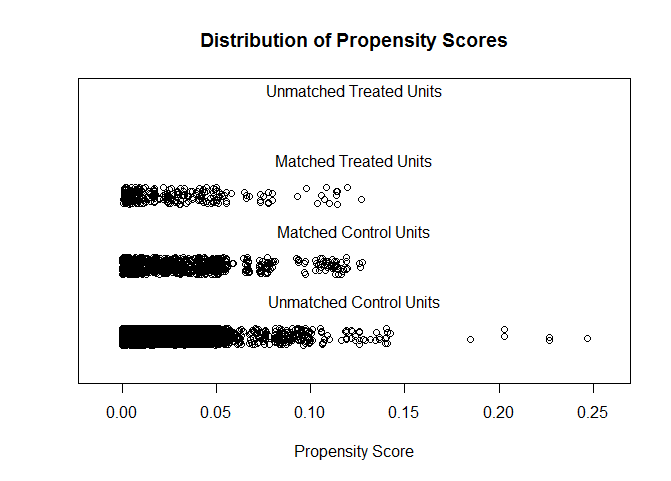


Table S2. Logistic Regression Model Used to Estimate Propensity Scores (Nearest Neighbour)

|  | Estimate | Std. Error | Statistic | p.value | OR | CI_lower | CI_upper |
| --- | --- | --- | --- | --- | --- | --- | --- |
| Intercept | -21.860 | 227.492 | -0.096 | 0.923 | 0.000 | 0.000 | 1.417751e+184 |
| Asian | 12.104 | 227.490 | 0.053 | 0.958 | 180546.201 | 0.000 | 7.937680e+198 |
| Black | 12.422 | 227.489 | 0.055 | 0.956 | 248176.574 | 0.000 | 1.090534e+199 |
| Mixed | 11.866 | 227.490 | 0.052 | 0.958 | 142401.342 | 0.000 | 6.260141e+198 |
| Refusal | 13.364 | 227.490 | 0.059 | 0.953 | 636532.685 | 0.000 | 2.799158e+199 |
| White | 12.547 | 227.489 | 0.055 | 0.956 | 281351.588 | 0.000 | 1.236127e+199 |
| Age | -0.005 | 0.008 | -0.588 | 0.557 | 0.995 | 0.980 | 1.011 |
| No qualification | -0.491 | 0.190 | -2.588 | 0.010 | 0.612 | 0.422 | 0.888 |
| Indeterminant | 1.510 | 0.148 | 10.227 | 0.000 | 4.527 | 3.389 | 6.046 |
| Young offender | 0.907 | 0.353 | 2.571 | 0.010 | 2.476 | 1.240 | 4.943 |
| OPD screened in | 0.927 | 0.154 | 6.000 | 0.000 | 2.526 | 1.866 | 3.420 |
| Age first convicted | -0.047 | 0.019 | -2.489 | 0.013 | 0.955 | 0.920 | 0.990 |
| Drugs ever misused | 3.502 | 1.006 | 3.479 | 0.001 | 33.177 | 4.615 | 238.525 |
| Some alcohol misuse problems | -0.011 | 0.189 | -0.060 | 0.952 | 0.989 | 0.683 | 1.431 |
| Significant alcohol misuse problems | 0.361 | 0.167 | 2.157 | 0.031 | 1.435 | 1.034 | 1.992 |
| History of self harm | 1.617 | 0.141 | 11.435 | 0.000 | 5.038 | 3.819 | 6.647 |
| OGRS | 0.002 | 0.004 | 0.526 | 0.599 | 1.002 | 0.995 | 1.009 |

PSM matching details:

Method: Nearest neighbour

Ratio: 4 controls per treated case

Distance: logistic regression propensity score

Replacement: no

Software: “MatchIt” R packages.

PSM Balance plots

Figure S3 Histogram plots


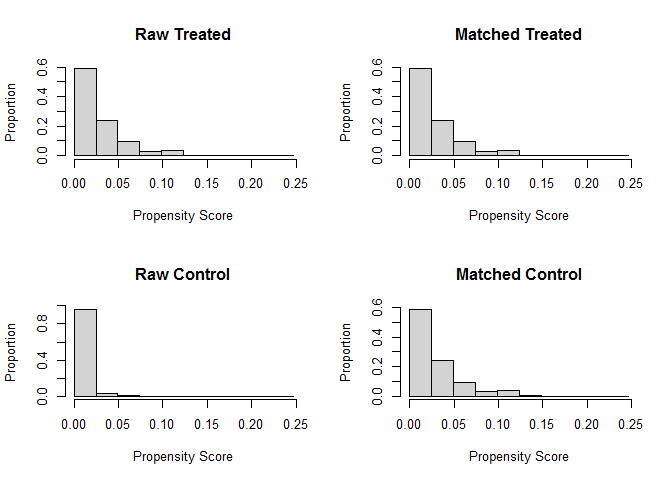


Figure S4. Jitter plots


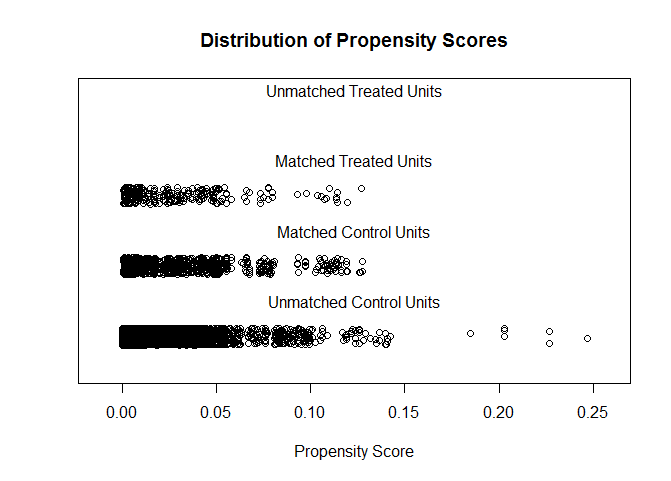


Sensitivity analysis: matching after eligibility restrictions

Sensitivity analyses were conducted to assess whether the timing of propensity score matching relative to eligibility restrictions influenced the results.

In the primary analysis, matching was conducted prior to linkage to NOMIS movement data, which identified individuals with open prison episodes during the study period. To assess the robustness of this approach, propensity score matching was repeated after restricting the dataset to individuals who met the final eligibility criteria (i.e., individuals with an open prison episode during the study period).

Optimal matching (1:1) was performed using the same covariates as the primary propensity score model.

Supplementary Table S3: Comparison of Primary and Sensitivity Analysis Results

|  | **Univariable** | | | **Adjusted*** | | |
| --- | --- | --- | --- | --- | --- | --- |
|  | **HR** | **95% CI** | **p** | **HR** | **95% CI** | **p** |
| ***Return to closed conditions*** | | | | | | |
| **Main analysis (n=638)** | 0.82 | 0.61 – 1.09 | .164 | 0.73 | 0.53 – 0.99 | .041 |
| **Sensitivity analysis (n=332)** | 1.23 | 0.87 – 1.74 | .249 | 1.23 | 0.86 – 1.77 | .263 |
| ***Release to the community*** | | | | | | |
| **Main analysis (n=638)** | 0.10 | 0.02 – 0.42 | .001 | 0.16 | 0.03 – 0.71 | .016 |
| **Sensitivity analysis (n=88)** | 1.40 | 0.22 – 8.85 | .723 | — | — | — |

**Note.** HR = hazard ratio; CI = confidence interval. PERS users are the reference group for all models. ***** Adjusted models include age, ethnicity (stratified), education, sentence type, OPD screening status, age at first conviction, alcohol misuse history, history of self-harm, and OGRS3 two-year reoffending score. Drugs misuse was additionally included in the primary adjusted model but excluded from the sensitivity adjusted model due to complete separation in the smaller sample. The adjusted sensitivity model for release to the community could not be reliably estimated due to only 4 events in the sensitivity sample (n = 88) and is therefore not reported (—). The sensitivity analysis used 1:1 optimal matching conducted after applying open prison eligibility criteria; the primary analysis used 4:1 matching conducted prior to eligibility criteria being applied.

Proportional hazard testing for survival analysis. P-values <.05 indicate the assumption of proportional hazards is violated.

Table S4. Unadjusted model (return to closed) proportional hazard test

|  | Chi-square | df | p-value |
| --- | --- | --- | --- |
| Full sample (N = 639) |  |  |  |
| Service | 4.51 | 1 | .034 |
| GLOBAL | 4.51 | 1 | .034 |
| Truncated at 500 days (N = 507) |  |  |  |
| Service | 3.01 | 1 | .083 |
| GLOBAL | 3.01 | 1 | .083 |

Table S5. Adjusted model (return to closed) proportional hazard test

|  | Chi-square | df | p-value |
| --- | --- | --- | --- |
| Full sample (N = 608) |  |  |  |
| Service | 5.98 | 1 | .015 |
| Age | 0.01 | 1 | .916 |
| Ethnicity | 13.6 | 4 | .009 |
| Education | <.01 | 1 | .976 |
| Sentence type | 4.52 | 2 | .010 |
| OPD screen | 0.03 | 1 | .861 |
| Age at first conviction | 3.60 | 1 | .058 |
| Drugs misuse | 0.40 | 1 | .530 |
| Alcohol misuse | 6.00 | 2 | .050 |
| History of self-harm | 0.02 | 1 | .878 |
| OGRS | 1.88 | 1 | .170 |
| GLOBAL | 34.8 | 16 | .004 |
| Truncated at 500 days (N = 507) |  |  |  |
| Service | 3.06 | 1 | .080 |
| Age | 0.23 | 1 | .633 |
| Ethnicity | 15.33 | 4 | .004 |
| Education | 0.07 | 1 | .782 |
| Sentence type | 2.66 | 2 | .265 |
| OPD screen | 0.06 | 1 | .811 |
| Age at first conviction | 7.29 | 1 | .007 |
| Drugs misuse | 0.01 | 1 | .935 |
| Alcohol misuse | 3.37 | 2 | .185 |
| History of self-harm | 0.86 | 1 | .352 |
| OGRS | 3.44 | 1 | .064 |
| GLOBAL | 34.51 | 15 | .005 |
| Truncated at 500 days and stratified by ethnicity (N = 507) |  |  |  |
| Service | 3.16 | 1 | .069 |
| Age | 0.26 | 1 | .613 |
| Education | 0.14 | 1 | .704 |
| Sentence type | 3.86 | 2 | .145 |
| OPD screen | 0.04 | 1 | .843 |
| Age at first conviction | 7.06 | 1 | .008 |
| Drugs misuse | <0.01 | 1 | .956 |
| Alcohol misuse | 2.82 | 2 | .244 |
| History of self-harm | 0.29 | 1 | .591 |
| OGRS | 3.83 | 1 | .050 |
| GLOBAL | 19.87 | 12 | .070 |

Note. Service use and ethnicity violate proportional hazard assumption suggesting a time-varying effect of these variables in the truncated to 500 days model. After stratifying for ethnicity, the proportional hazard assumption is no longer violated.

Table S6. Unadjusted model (release to the community)

|  | Chi-square | df | p-value |
| --- | --- | --- | --- |
| Full sample (N = 638) |  |  |  |
| Service | 8.66 | 1 | 0.003 |
| GLOBAL | 8.66 | 1 | 0.003 |
| Truncated post 150 days (N = 407) |  |  |  |
|  | Chi-square | df | p-value |
| Service | <.01 | 1 | 1 |
| GLOBAL | <.01 | 1 | 1 |

Table S7. Adjusted model (release to the community)

|  | Chi-square | df | p-value |
| --- | --- | --- | --- |
| Full sample (N = 638) |  |  |  |
| Service | 6.49 | 1 | .011 |
| Age | 0.01 | 1 | .920 |
| Ethnicity | 12.49 | 4 | .014 |
| Education | 2.72 | 1 | .100 |
| Sentence type | 10.37 | 2 | .005 |
| OPD screen | 0.02 | 1 | .88 |
| Age at first conviction | 3.60 | 1 | .058 |
| Drugs misuse | 0.40 | 1 | .530 |
| Alcohol misuse | 6.00 | 2 | .050 |
| History of self-harm | 0.02 | 1 | .878 |
| OGRS | 1.88 | 1 | .170 |
| GLOBAL | 34.8 | 16 | .004 |
| Truncated at post 150 days (N = 507) |  |  |  |
| Service | 3.06 | 1 | .080 |
| Age | 0.24 | 1 | .628 |
| Ethnicity | 15.34 | 4 | .004 |
| Education | 0.06 | 1 | .804 |
| Sentence type | 2.67 | 2 | .263 |
| OPD screen | 0.08 | 1 | .783 |
| Age at first conviction | 7.29 | 1 | .007 |
| Drugs misuse | 0.01 | 1 | .935 |
| Alcohol misuse | 3.37 | 2 | .185 |
| History of self-harm | 0.87 | 1 | .352 |
| OGRS | 3.44 | 1 | .064 |
| GLOBAL | 34.51 | 16 | .005 |
| Truncated post 150 days and stratified by ethnicity (N = 507) |  |  |  |
| Service | 0.01 | 1 | .908 |
| Age | 0.01 | 1 | .945 |
| Education | 0.18 | 1 | .671 |
| Sentence type | 1.60 | 2 | .450 |
| OPD screen | 3.11 | 1 | .577 |
| Age at first conviction | 6.46 | 1 | .011 |
| Drugs misuse | 2.41 | 1 | .121 |
| Alcohol misuse | 1.15 | 2 | .563 |
| History of self-harm | 1.67 | 1 | .196 |
| OGRS | 1.14 | 1 | .285 |
| GLOBAL | 14.96 | 12 | .243 |

Covariate balance between the PERS and matched non-PERS group were assessed using standardised mean differences (SMD). The matching weights were applied to account for the variable number of controls per treated cases. SMD <.02 indicate acceptable balance. Most covariates are well balanced, apart from sentence types and age at first conviction, which still show improvements in reduced SMD compared to the pre-matched sample.

Table S8. Balance measures for pre-post matched sample

| Covariate | Pre-matched sample SMD | post-matched sample weighted SMD |
| --- | --- | --- |
| Age | -0.463 | -0.084 |
| Ethnicity |  |  |
| Asian | -0.169 | -0.022 |
| Black | -0.061 | 0.010 |
| Mixed | -0.117 | 0.002 |
| Refused | 0.058 | 0.024 |
| White | 0.324 | 0.034 |
| Education (no qualifications) | -0.122 | -0.027 |
| Sentence type |  |  |
| Determinate | -0.925 | -0.218 |
| Indeterminate | -0.882 | 0.247 |
| Young offender | 0.079 | -0.029 |
| OPD screen (screened in) | 0.413 | 0.0877 |
| Age at first conviction | -0.998 | -0.236 |
| Drugs ever misused (misused) | 3.626 | 0.003 |
| Alcohol misuse |  |  |
| No problems | -0.615 | 0.073 |
| Some problems | -0.085 | 0.011 |
| Significant problems | 0.610 | 0.084 |
| History of self-harm | 1.096 | 0.011 |
| OGRS | 0.511 | 0.039 |
