# Supplementary material for: Evaluating outcomes of psychological support services in minimum security prisons in England: a quasi-experimental retrospective cohort study using propensity score matching and survival analysis
Source: BMJ Open. 2026 Mar 30;16(3):e110540. doi: 10.1136/bmjopen-2025-110540 (PMC13052529; doi:10.1136/bmjopen-2025-110540)
Supplement: Supplementary file 2 [file bmjopen-2025-110540.reviewer_comments.pdf]

PEER REVIEW HISTORY

BMJ Open publishes all reviews undertaken for accepted manuscripts. Reviewers are asked to complete a checklist review form and are provided with free text boxes to elaborate on their assessment. These free text comments are reproduced below.

ARTICLE DETAILS

Title (Provisional)

Evaluating outcomes of psychological support services in minimum security prisons in England: a quasi-experimental retrospective cohort study using propensity score matching and survival analysis

Authors

Mathlin, Georgina; Jones, Hannah; Lewis, Carine; Cooper, Claudia; Freestone, Mark

---

VERSION 1 - REVIEW

**Reviewer**	**1**
**Name**	**Kelson, Zoe**
**Affiliation**	**University of Exeter, Mathematics**
**Date**	**28-Oct-2025**
**COI**	**None**

This study aims to test the hypotheses that offenders in minimum security prisons in England, identified as having a likely personality disorder and high reoffending risk, who opted in to psychologically informed support (Pathways Enhanced Resettlement Service; PERS), were (a) less likely to return to more restrictive closed prison conditions, and (b) released to the community sooner, compared to matched non-PERS users.

Reviewer comments:

"We analysed routine PERS and UK Ministry of Justice administrative data, for all clients enrolled on PERS between 1.4.2019 until 30.4.2022"

Can the authors please confirm if the data used in the study provides complete coverage?

"All adult males who joined PERS from 01.04.2019 (the data first service opened) until 30.04.2022 were included in the 'PERS users' group. "

Can the authors please comment on whether the analysed cohort can be considered to be representative?

Can the fact that only males are included please be acknowledged in the discussion of the study limitations?

"All new open prison arrivals with indeterminant sentences or deemed at high risk of self-harm are screened against standard criteria by PERS staff (20); those at 'high-risk' category of reoffending; who have been at risk of self-harm or suicide in the last 12 months or who have previously been returned from open prison or the community to closed conditions are invited by a staff member to join PERS, which is voluntary."

Whilst the authors have attempted to match on criteria for the 'high-risk' category of reoffending, can they please further demonstrate that they have matched on the propsensity to join PERS (i.e. accept the invite)?

"propensity score matched control group of non-PERS users"

and

"A control group of 'non-PERS users' (adult males entering any open prison between 01.04.2019 until 30.04.2022 that had never used PERS, including those who were approached but declined) were identified using Propensity Score Matching (PSM). The MatchIt (25) and optmatch (26) R packages were used. Optimal matching was chosen as it minimises the average absolute distance across all matched pairs. We selected covariates for the PSM to align with PERS eligibility criteria and baseline characteristics."

A rigorous matching methodology has been applied.

Can the PSM model please be shown in full in the supplementary material?

"We compared missing and non-missing data and found a significant difference across each covariate, suggesting the missing data was dependent upon another observed variable in the dataset and was considered to be missing at random (MAR; Rubin, 1976). Missing Imputation Chained Equations (MICE) were used with the R package mice (28) to impute missing data for all variables with missingness apart from ethnicity, as this is a characteristic not considered suitable for imputation. Logistic regressions were used to impute the binary variables (education, drugs ever misused), polytomous logistic regressions for the categorical variables (alcohol misuse in the past) and predictive mean matching for the continuous variables (age at first conviction, OGRS score). The imputation was checked, and all imputed data followed the same distribution as the original data"

Can the authors please clarify how much data was missing for each variable?

Can a sensitivity analysis (e.g. a complete case analysis) please be conducted to help demonstrate the robustness of the study findings to missing data?

"A four to one ratio of matching was used, identifying four non-PERS service users for each PERS service user. This oversampling was designed to ensure that the control group included a sufficient sample in the Cox Proportional hazard models as the matching took place prior to individuals being identified as having been in an open prison and therefore the final number of controls to service user ratio was less than four to one. "

and

"Out of the 1076 individuals identified through the matching, only 358 had been in an open prison between April 2019 and April 2022, so this was the final sample size of the matched control group."

To clarify, some PERS service users are matched to more/less non-PERS service users than others in the analysis?

This imbalance might incur confounding by oversampling some non-PERS service users and undersampling others?

Can a consistent matching ratio please be re-applied prior to analysis (i.e. 1:1)?

"Differences between the groups were explored using appropriate univariate tests."

Can these tests please be specified?

"We explored whether the rate of being returned to closed conditions prison and released into the community differed between PERS users and the matched group of offenders who had not used PERS using Cox Proportional Hazard models using the survminer R package (29)"

Can models please include covariates to adjust for any residual confounding?

"The models were clustered by the individual to account for people who were in PERS multiple times throughout the study period"

Can the authors please clarify how these repeat PERS users were accounted for in the matching methodology?

How many PERS service users were in PERS multiple times, and what is the average number of times in PERS for each service user?

Can the authors please explore a sensitivity analysis to only analyse instances of first PERS use?

"The initial models (N = 638) violated the proportional hazard test (see supplementary materials), indicting an inconsistent effect of PERS service use and type of open prison over time on return to closed conditions and release into the community. The model exploring the effect of PERS use on risk of return to closed conditions were re-run only including the first 500 days after entering open prison and the proportional hazard test was not violated, therefore these models are reported. The model investigating the effect of PERS on release into the community were re-run looking at 150 days after entering the open prison. The proportional hazard assumption was no longer violated and therefore these models are reported."

In addition to this redefined and restricted analysis, can stratification of the variable causing the violation of the proportional hazards assumption please be considered for Cox regression models to be re-run?

"We compared 258 PERS users and 358 controls, matched for ethnicity, age at first offence and sentencing, education, sentence type, personality disorder screening, risk of reoffending scores, and historical drug and alcohol use."

Can pre- and post- matching standardised mean differences (SMDs) please be reported and tabulated?

"there was a trend towards a lower likelihood of return to closed conditions prisons (HR = 0.84, 95% CI 0.62-1.12) than matched controls. " [Abstract]

and

"PERS service use was associated with a reduced rate of return to closed conditions (p = .226, HR = .84, 95% CI 0.62-1.12)."

Can the authors please refrain from inferring a 'reduced rate' or a 'trend towards' for statistically non-significant outcomes (rather, an association has not been evidenced here)?

"Figure 1. Kaplan-Meier Curve PERS vs non-PERS release into the community"

and

"The Kaplan-Meier Curve (Figure 2) shows the probability of being returned to closed conditions across 1200 days by service use. "

Can this methodology please be specified in the Statistical Analysis section?

"Some limitations of this study should be considered"

A discussion on the study limitations is provided.

Thanks for providing a copy of the STROBE checklist.

Reviewer 2

Name Stoliker, Bryce E.

Affiliation University of Saskatchewan, Centre for Forensic Behavioural Science and Justice Studies

Date 06-Nov-2025

COI None

Abstract & Highlights

- Pg. 2, line 43: The authors have a section in the Abstract, “Clinical Applications,” that’s blank.

- Pg. 3, line 34-41: The authors note the need for further research, but what does the study add itself in terms of research, policy, or practice? How does it advance knowledge or efforts in the areas of research, policy, practice?

Introduction

- The Introduction section provides a general overview of the key focuses, particularly, highlighting the nature of minimum security prisons and their purpose, the issue of persons living with personality disorder and the challenges surrounding the rehabilitation of these individuals, and a brief note on the psychologically informed services (OPDP and PERS) to assist with addressing the needs of persons living with personality disorder. However, there are some revisions that could be made to enhance this section and set the study up with stronger introductory material. First, while more of a cosmetic revision, discussion around the issues of (i) personality disorders and (ii) minimum security prisons can be better integrated. It reads as two disconnected discussions. Second, considering the focus of the study is on criminal justice outcomes (i.e., return to closed custody and community resettlement), this should be emphasized a little more earlier on. Third, it would help to more clearly operationalize the outcomes of interest for this study so it's clear that the programming is assessed according to individuals' move from minimum back to closed custody or earlier release to community. Fourth, there's very little information about the PERS, which is the program that is effectively being assessed. What exactly is the treatment dosage (i.e., length of involvement, types of services/activities provided to address personality disorder and enhance release to community, frequency of sessions, etc.) and what components aim to assist those with personality disorders and how? Fifth, and relatedly, has this program been formally evaluated? If so, include some findings from the evidence-based evaluation. If not, note this. Sixth, the final paragraph is setting up the entire focus of the study but is quite limited. For instance, given the lack of information on PERS and how it intends to aid in the rehabilitation of the target population, it's not easy to make the connection between the programming and the expected outcomes. Seventh, the study includes time-based data and survival analysis, so why not include research questions or hypotheses that address the time-based element of the methodology? Indeed, on pg. 7, line 17-24, it is indicated that the primary outcomes were return to closed prison and release to community but focused on the time element of these outcomes.

Method

- Study design and data sources: Generally speaking, this subsection is clear and well-written. It would be helpful to just make clear any distinctions between the collection and use of data between PERS and non-PERS study participants. What differences and similarities exist in terms of pulling from the noted data sources? In addition, were the data pulled from the data sources and merged in a research data base? Further, it would help to clarify the study period in this subsection. Finally, while it is noted that it is a retrospective cohort study, the authors should clarify that they collected and analyzed secondary data available through the various sources and that no primary data were collected.

- Study participants: This subsection could include description of both the treatment and control groups for ease of discussion and description of the comparison groups. With

respect to the description of the treatment group (PERS), it's unclear exactly how people come to this program. In the introduction, it seemed like it was an element of moving security classifications/prison and preparing for release, whereas pg. 6 suggests it begins at prison entry (or re-entry) and for a select population (high risk of offending, self-harm, etc.). In general, the authors should more clearly describe this program and its target population, how people begin the programming. In addition, there should be a more fulsome description of the study participants within the treatment group. For instance, how were participants selected into the study, how many were selected into the study, were any excluded and why, what was the pool of PERS users? There should be a systematic description of who qualifies for PERS and how PERS users were included into the study. Include the number of participants.

- Control group: As above, integrate into a single section on study participants. Further detail should be provided on the pool of control group participants and whether any were excluded prior to propensity score matching. It is noted that PSM was based, among other things, on PERS eligibility criteria. This criteria should be outlined earlier. Again, this relates back to a need for a more fulsome description of the program, what it entails, and who it targets. Further, on pg. 6, when describing the covariates, the authors should separate and note the eligibility criteria vs. other characteristics. Pg. 6, line 47-57 discusses missing data analysis and management. Is this missing data for all study participants or the control group only? Who did the authors compare missing and non-missing data for, or was it a generally assessment of missingness across each covariate for the total sample? More detail is needed on what the missing data analysis consisted of and how it was concluded that data were MAR. Was Little's MCAR used to first assess the nature of missingness, as this is the first step before tests for MAR or MNAR. Did imputation consist of a single imputation or multiple imputation, with the latter being more robust? Finally, it is not entirely clear why a four to one ratio of matching was used. Further details and justification are needed to explain this approach. The current explanation is confusing. Include the number of participants.

- Outcomes: Would there be any need to left censor data to account for outcomes experienced before the study period (1.4.2019), or is the start of the study period the point at which all participants began open custody? How were data controlled for exposure time? For instance, accounting for people in open prison well before or well after the start of the study period? In terms of censoring, only right censoring was done?

- Statistical Analysis: Kaplan-Meier curves are presented, why not include note of these analyses here as well? Were confounding factors controlled in the Cox Regression models? If so, which. If not, why?

- Throughout the manuscript, the authors talk about "rate" of return to closed conditions and release into the community, when in reality they're discussing Hazard Ratios and probability across time which would be interpreted most appropriately as the likelihood or

probability of experiencing one of the key events and experiencing it quicker than the referent group.

- Involvement of people with lived experience in the study: Why was this individual involved and what role did they place exactly? How did it benefit the study?

- It would be beneficial to know more about the setting(s) in which the study took place, such as prison characteristics, especially for international readership.

Results

- If 11 people were missing data and were removed, leaving 258 individuals in the study, why were the 269 individuals matched to a control group? The treatment group should be defined prior to matching and only use those involved in the study, not those excluded. What's the justification for the approach taken by the authors? It's also curious the approach taken to define/identify the control group. Why not establish those who had been in an open prison during the study period first, then use PSM to match with the treatment group. So, did the authors match the treatment group with all possible people in custody that had not used PERS? It would seem most appropriate to first identify any person in open custody who had not used PERS as the first step, rather than taking the deductive approach the authors used here. Typically, with PSM there are equal numbers in the treatment and control group. Related to an above point, why is this not the case for the current study?

- With the descriptive statistics and Table 1, the authors effectively introduced a new outcome measure into the study. It appears they are not only interested in returns to closed custody and release to the community, but also maintaining stay in open prison. So, why is this not included as part of the larger study/analysis and not simply a descriptive factor?

- Table 1: for sentence type, "young offender" is included. The study notes a focus on adults, so who would qualify as a young offender?

- Pg. 9: Who were the 407 people used in the analysis to assess release to the community and how are they grouped and how many? Further, what does it mean when the authors say they are analyzing data after 150 days only for the release to community analysis?

- Pg. 10: The following sentence is inaccurate in terms of interpretation: "PERS service users had a 48% reduced rate of being released to the community compared to the non-PERS sample" … with hazard and odds ratios, it's best to invert those ratios below 1.0 (by 1/HR) to get a more meaningful interpretation. Here, it would mean that PERS services users were 1.92 times less likely to be release to the community.

- Pg. 10: Who were the 507 people used in the analysis to assess return to closed custody and how are they grouped and how many? Further, what does it mean when the authors say they are analyzing data in the first 500 days? Also, improve interpretation of odds/hazard ratios below 1.0 as above.

- For the Cox proportional hazard models, are PERS users the target category vs. the reference category of non-service users? This should be noted to avoid any confusion.

Discussion

- The information presented in the Discussion section is generally fine, but it is quite brief and limited. It would benefit from reflecting more on the current findings and drawing deeper comparisons to previous research, especially research that has been conducted on the PERS program. It would also benefit to discuss more about what future research could/should do to assess the PERS program based on the current findings, with pointed recommendations. This seems to be a big implication of the current study, where it would drive more research than provide answers into its efficacy. So this should be leveraged and discussed in greater detail, again, with pointed recommendations.

VERSION 1 - AUTHOR RESPONSE

Comment	Response
We analysed routine PERS and UK Ministry of Justice administrative data, for all clients enrolled on PERS between 1.4.2019 until 30.4.2022"<br>Can the authors please confirm if the data used in the study provides complete coverage?	We confirm that the data used in the study provides complete coverage. The analysis includes all clients enrolled on PERS from 1 April 2019 to 30 April 2022, with full corresponding Ministry of Justice administrative records. There were no known gaps or exclusions in the datasets for this period. We have made this clear within the study design and data sources section of the manuscript.
"All adult males who joined PERS from 01.04.2019 (the data first service opened) until 30.04.2022 were included in the 'PERS users' group. "<br>Can the authors please comment on whether the analysed cohort can be considered to be representative?<br>Can the fact that only males are included please be acknowledged in the discussion of the study limitations?	This represents complete coverage of the population and therefore is representative of all service users during this period. We have added in this sentence in the study participants and setting section to clarify that the complete cohort has been analysed and is therefore representative of all PERS users during the study period. PERS is only available in the male prison estate, and this is

	why only males were included. We have added further detail into the introduction explain this.
"All new open prison arrivals with indeterminant sentences or deemed at high risk of self-harm are screened against standard criteria by PERS staff (20); those at 'high-risk' category of reoffending; who have been at risk of self-harm or suicide in the last 12 months or who have previously been returned from open prison or the community to closed conditions are invited by a staff member to join PERS, which is voluntary."<br>Whilst the authors have attempted to match on criteria for the 'high-risk' category of reoffending, can they please further demonstrate that they have matched on the propensity to join PERS (i.e. accept the invite)?	We aimed to identify a matched control group who were eligible for PERS but either were not offered it (being in an open prison without the service or invited to join but did not accept). We used baseline demographic characteristics and all available variables that map onto eligibility for PERS.
Can the PSM model please be shown in full in the supplementary material?	Thank you for raising this point. We have now included as supplementary material, the PSM model, details of the matching and the histogram and jitter plots.
"We compared missing and non-missing data and found a significant difference across each covariate, suggesting the missing data was dependent upon another observed variable in the dataset and was considered to be missing at random (MAR; Rubin, 1976). Missing Imputation Chained Equations (MICE) were used with the R package mice (28) to impute missing data for all variables with missingness apart from ethnicity, as this is a characteristic not considered suitable for imputation. Logistic regressions were used to impute the binary variables (education, drugs ever misused), polytomous logistic regressions for the categorical variables (alcohol misuse in the past) and predictive mean matching for the continuous variables (age at first conviction, OGRS score). The imputation was checked, and all imputed data followed the same distribution as the original data"<br>Can the authors please clarify how much data was missing for each variable?	Thank you for pointing out that we had not included this information. We have included an overall percentage of missingness across the dataset as it would impact the readability to report each variable and the percentage of missingness.
Can a sensitivity analysis (e.g. a complete case analysis) please be conducted to help demonstrate the robustness of the study findings to missing data?	We have explored whether a complete case analysis is feasible. There was no

	missing outcome data in this study. The reported 24% of missing data occurred entirely in covariates used for propensity score matching, not in the outcome time-to-event variables.<br><br>We conducted a complete case restriction across all matching covariates; however, this resulted in a substantially reduced sample, including only nine individuals in the PERS group. This sample size was insufficient to perform propensity score matching or to meaningfully compare complete-case and imputed analyses. Consequently, a complete-case sensitivity analysis was not feasible for the matched analysis.<br><br>We have clarified the nature and location of missing data and added this as a limitation in the Discussion section.
"A four to one ratio of matching was used, identifying four non-PERS service users for each PERS service user. This oversampling was designed to ensure that the control group included a sufficient sample in the Cox Proportional hazard models as the matching took place prior to individuals being identified as having been in an open prison and therefore the final number of controls to service user ratio was less than four to one. "<br>and<br>"Out of the 1076 individuals identified through the matching, only 358 had been in an open prison between April 2019 and April 2022, so this was the final sample size of the matched control group."<br>To clarify, some PERS service users are matched to more/less non-PERS service users than others in the analysis?<br>This imbalance might incur confounding by oversampling some non-PERS service users and	Thank you for raising this important point. The varying matching rate in the final sample represents post-matching attrition due to issues with linking the data to individuals in open prisons. If a 1:1 matching ratio was used a substantial loss of PERS users may no longer have eligible matches and therefore this would have reduced the sample size, increased bias through selection exclusion and reduced the representativeness of the exposure group.

undersampling others?<br>Can a consistent matching ratio please be re-applied prior to analysis (i.e. 1:1)?	To address the concerns about imbalance we have re-calculated balance diagnostics on the final linked and matched sample, demonstrating that covariates remain well balanced and present this in the supplementary materials alongside including the covariates within an adjusted cox models to account for residual confounding. We have also added in the discussion of limitations that an inconsistent matching ratio may have introduced confounding.
"Differences between the groups were explored using appropriate univariate tests."<br>Can these tests please be specified?	We have specified the tests within the statistical analysis section of the methods.
"We explored whether the rate of being returned to closed conditions prison and released into the community differed between PERS users and the matched group of offenders who had not used PERS using Cox Proportional Hazard models using the survminer R package (29)"<br>Can models please include covariates to adjust for any residual confounding?	Thank you for this suggestion. We have now run the models with the covariates, reporting the unadjusted and adjusted models in the results.
"The models were clustered by the individual to account for people who were in PERS multiple times throughout the study period"<br>Can the authors please clarify how these repeat PERS users were accounted for in the matching methodology?	Individuals could enter PERS more than once during the study period, and our matching approach treated each PERS entry as a separate treatment episode. Therefore, a person with multiple PERS episodes could be matched multiple times, each episode matched to controls based on covariates measured at that time. Because these repeated episodes are not independent, the Cox models were clustered by individual ID to adjust for within-person correlation. We have clarified

	this approach within the Methods section.
How many PERS service users were in PERS multiple times, and what is the average number of times in PERS for each service user?	We have clarified in the methods section that repeated cases were across entry into the open prison, rather than isolated within the PERS group. 69 individuals across the data (both PERS and non-PERS) were in open prison more than once across the study period, 29 of those were in PERS more than once during the study period. The average was two entries to the open prison. We have added this detail on repeated entries into the statistical analysis section of the methods.
Can the authors please explore a sensitivity analysis to only analyse instances of first PERS use?	The subset of data we have available does not contain information on entry dates to open prison and therefore we cannot be certain which episode is the individuals first to be able to do this. We have added a sentence into the limitations to acknowledge this.
"The initial models (N = 638) violated the proportional hazard test (see supplementary materials), indicting an inconsistent effect of PERS service use and type of open prison over time on return to closed conditions and release into the community. The model exploring the effect of PERS use on risk of return to closed conditions were re-run only including the first 500 days after entering open prison and the proportional hazard test was not violated, therefore these models are reported. The model investigating the effect of PERS on release into the community were re-run looking at 150 days after entering the open prison. The proportional hazard assumption was no longer violated and therefore these models are reported."<br>In addition to this redefined and restricted analysis, can stratification of the variable causing the violation	We re-run an adjusted model stratified by ethnicity, the variable causing the violation. For the unadjusted model, stratifying by the variable causing the violation (service use) would mean not obtaining HR for the variable, and as this is the key analytical variable we felt this was not a feasible option. We have clarified the number of restricted and stratified models run within the statistical analysis section of the methods.

of the proportional hazards assumption please be considered for Cox regression models to be re-run?	
"We compared 258 PERS users and 358 controls, matched for ethnicity, age at first offence and sentencing, education, sentence type, personality disorder screening, risk of reoffending scores, and historical drug and alcohol use."<br>Can pre- and post- matching standardised mean differences (SMDs) please be reported and tabulated?	We have presented this within the supplementary materials.
"there was a trend towards a lower likelihood of return to closed conditions prisons (HR = 0.84, 95% CI 0.62-1.12) than matched controls. " [Abstract]<br>and<br>"PERS service use was associated with a reduced rate of return to closed conditions (p = .226, HR = .84, 95% CI 0.62-1.12)."<br>Can the authors please refrain from inferring a 'reduced rate' or a 'trend towards' for statistically non-significant outcomes (rather, an association has not been evidenced here)?	Thank you. Non-significant has been emphasised in both the abstract and the results where this is relevant.
"Figure 1. Kaplan-Meier Curve PERS vs non-PERS release into the community"<br>and<br>"The Kaplan-Meier Curve (Figure 2) shows the probability of being returned to closed conditions across 1200 days by service use. "<br>Can this methodology please be specified in the Statistical Analysis section?	Thank you, a sentence explaining the step of generating Kaplan-Meier Curves has been included in the Statistical Analysis section.
- Pg. 2, line 43: The authors have a section in the Abstract, “Clinical Applications,” that’s blank.	That has been removed.
Pg. 3, line 34-41: The authors note the need for further research, but what does the study add itself in terms of research, policy, or practice? How does it advance knowledge or efforts in the areas of research, policy, practice?	Thank you for suggesting some further emphasis on this. We have added this sentence: “This study provides useful information for policymakers about how PERS influences lengths of stays in open prisons; it lengthens them and reduces the likelihood of return to closed conditions. Given the economic and potential wellbeing benefits of open versus closed prisons, this evidence could support

	further investment in PERS services" into the conclusion.
First, while more of a cosmetic revision, discussion around the issues of (i) personality disorders and (ii) minimum security prisons can be better integrated. It reads as two disconnected discussions	Thank you for raising this. The second and third paragraph have been integrated which we think integrates these two discussions more effectively.
Second, considering the focus of the study is on criminal justice outcomes (i.e., return to closed custody and community resettlement), this should be emphasized a little more earlier on.	Thank you for pointing this out. We have emphasised the focus on these transitions in the third paragraph.
Third, it would help to more clearly operationalize the outcomes of interest for this study so it's clear that the programming is assessed according to individuals' move from minimum back to closed custody or earlier release to community.	Relatedly to the above, the emphasis on the criminal justice outcomes has further made clear these transitions are an aim of the PERS.
Fourth, there's very little information about the PERS, which is the program that is effectively being assessed. What exactly is the treatment dosage (i.e., length of involvement, types of services/activities provided to address personality disorder and enhance release to community, frequency of sessions, etc.) and what components aim to assist those with personality disorders and how?	Thank you for highlighting this. Further detail about PERS, typical length and amount of involvement has been included alongside more clear statements of how the programme aims to assist those with personality disorder through the activities available to individuals.
Fifth, and relatedly, has this program been formally evaluated? If so, include some findings from the evidence-based evaluation. If not, note this.	This is the first formal evaluation of PERS and so this has been more clearly noted.
Sixth, the final paragraph is setting up the entire focus of the study but is quite limited. For instance, given the lack of information on PERS and how it intends to aid in the rehabilitation of the target population, it's not easy to make the connection between the programming and the expected outcomes	We have added in some further context to the link between the PERS programming and its intended outcomes in this final paragraph.
Seventh, the study includes time-based data and survival analysis, so why not include research questions or hypotheses that address the time-based element of the methodology? Indeed, on pg. 7, line 17-24, it is indicated that the primary outcomes were return to closed prison and release to community but focused on the time element of these outcomes.	Thank you for raising this. We have amended the language of the hypothesis to highlight the time-based element that is of interest in the study using survival analysis.
It would be helpful to just make clear any distinctions between the collection and use of data between PERS and non-PERS study participants.	We have added further detail into this section to make it clear where there were

What differences and similarities exist in terms of pulling from the noted data sources? In addition, were the data pulled from the data sources and merged in a research data base? Further, it would help to clarify the study period in this subsection. Finally, while it is noted that it is a retrospective cohort study, the authors should clarify that they collected and analyzed secondary data available through the various sources and that no primary data were collected.	differences in pulling data for PERS and non-PERS participants. We have made it clearer the stages of the datasets being linked.<br>We have clarified this study only used secondary data.
This subsection could include description of both the treatment and control groups for ease of discussion and description of the comparison groups. With respect to the description of the treatment group (PERS), it's unclear exactly how people come to this program. In the introduction, it seemed like it was an element of moving security classifications/prison and preparing for release, whereas pg. 6 suggests it begins at prison entry (or re-entry) and for a select population (high risk of offending, self-harm, etc.). In general, the authors should more clearly describe this program and its target population, how people begin the programming. In addition, there should be a more fulsome description of the study participants within the treatment group. For instance, how were participants selected into the study, how many were selected into the study, were any excluded and why, what was the pool of PERS users? There should be a systematic description of who qualifies for PERS and how PERS users were included into the study. Include the number of participants.	We have moved the information about how individuals enter the service into the introduction. The further detail about the service added into the introduction should make it clearer who the target population is.<br>This study represents the full population of PERS service users during the study period and clarification of the complete coverage has been added into the study participants section. We have included the number of participants in this section.
Control group: As above, integrate into a single section on study participants. Further detail should be provided on the pool of control group participants and whether any were excluded prior to propensity score matching. It is noted that PSM was based, among other things, on PERS eligibility criteria. This criteria should be outlined earlier.	We have removed the control group sub-heading to have one 'Study participants' section.<br>We have included a clarification of the pool of control group participants and that there were no exclusions prior to the PSM. The eligibility criteria for PERS has now been included within the introduction.
Further, on pg. 6, when describing the covariates, the authors should separate and note the eligibility criteria vs. other characteristics	Thank you this is a helpful point that increases the clarity. The baseline

	characteristics and eligibility criteria covariates (indeterminant sentences, self-harm, high-risk of reoffending) have been separated.
Is this missing data for all study participants or the control group only? Who did the authors compare missing and non-missing data for, or was it a generally assessment of missingness across each covariate for the total sample? More detail is needed on what the missing data analysis consisted of and how it was concluded that data were MAR. Was Little's MCAR used to first assess the nature of missingness, as this is the first step before tests for MAR or MNAR.	The missing data is for the full data set of PERS and non-PERS (baseline demographics and covariates). We have added some detail into the paragraph on missing data within the study participants and settings section in the methods to make this clearer. We appreciate that Little's MCAR test can be used to assess whether data are consistent with MCAR. We used the approach of evaluating missingness patterns visually and then descriptively, as described by Tierney & Cook (2023). Further detail on this process has been added for clarity within the study participants and setting section in the methods.
Did imputation consist of a single imputation or multiple imputation, with the latter being more robust?	The imputation was multiple with m = 5. This detail has been added into the paragraph describing the imputation in the methods.
Finally, it is not entirely clear why a four to one ratio of matching was used. Further details and justification are needed to explain this approach. The current explanation is confusing. Include the number of participants.	Further details have been included in the study participants and setting section of the methods about the data linking approach and when the matching took place to justify the 4:1 ratio matching. This whole paragraph has been re-organised to clarify the order of the matching vs identifying whether the control group were in open prison and

	therefore the need to oversample the control group.
Would there be any need to left censor data to account for outcomes experienced before the study period (1.4.2019), or is the start of the study period the point at which all participants began open custody?	Participants may have been in the open prison before the study period but they were not receiving PERS, the exposure of interest at the start of the study period was the commencement of PERS services, so we have not left censored.
How were data controlled for exposure time? For instance, accounting for people in open prison well before or well after the start of the study period? In terms of censoring, only right censoring was done?	Exposure time was defined from the start of the study period (1.4.2019) until the occurrence of the event of interest or until censoring. Only right censoring was applied; participants were censored at the end of the study period or upon exit from open custody, whichever occurred first. Time spent in open custody prior to 1.4.2019 was not included in the risk period, as the exposure was PERS.
Kaplan-Meier curves are presented, why not include note of these analyses here as well? Were confounding factors controlled in the Cox Regression models? If so, which. If not, why?	We have included a note of the KM analyses in the text (statistical analysis section of methods), but the inferential results come from the Cox models and so detailed numerical estimated from the KM have not been reported in-text. We have reported unadjusted and adjusted models where confounding factors are controlled for.
Throughout the manuscript, the authors talk about “rate” of return to closed conditions and release into the community, when in reality they’re discussing Hazard Ratios and probability across time which would be interpreted most appropriately as the likelihood or probability of experiencing one of the key events and experiencing it quicker than the referent group	We have amended the use of the term ‘rate’ to likelihood throughout the manuscript to increase the interpretability of the hazard ratios.

Involvement of people with lived experience in the study: Why was this individual involved and what role did they place exactly? How did it benefit the study?	Further detail about this individual, their involvement and their contribution has been added to this section.
It would be beneficial to know more about the setting(s) in which the study took place, such as prison characteristics, especially for international readership.	We have provided some further information on the open prison settings and PERS in the methods section.
- If 11 people were missing data and were removed, leaving 258 individuals in the study, why were the 269 individuals matched to a control group? The treatment group should be defined prior to matching and only use those involved in the study, not those excluded.	Thank you for pointing this out. We apologise, this was an error in the write up. Only the individuals with the complete data (n = 258) were matched to the control group.
What's the justification for the approach taken by the authors? It's also curious the approach taken to define/identify the control group. Why not establish those who had been in an open prison during the study period first, then use PSM to match with the treatment group. So, did the authors match the treatment group with all possible people in custody that had not used PERS? It would seem most appropriate to first identify any person in open custody who had not used PERS as the first step, rather than taking the deductive approach the authors used here. Typically, with PSM there are equal numbers in the treatment and control group. Related to an above point, why is this not the case for the current study?	We were constrained by the data availability and data sharing processes which meant the matching took place prior to being able to identify whether individuals in the control group were in open prison. We have emphasised this more clearly in the manuscript, and provided details within the limitations section about this approach.
With the descriptive statistics and Table 1, the authors effectively introduced a new outcome measure into the study. It appears they are not only interested in returns to closed custody and release to the community, but also maintaining stay in open prison. So, why is this not included as part of the larger study/analysis and not simply a descriptive factor?	Remaining in open prison is implicitly included in the survival models. In the Cox proportional hazards framework, the event of interest is either return to closed custody or release to the community. Participants who experience neither event during the study period are right-censored, meaning they remained in open custody throughout follow-up. Therefore, maintaining stay in open prison is not analysed as a separate outcome but is reflected in the risk set and

	censoring structure of the survival model.
- Table 1: for sentence type, "young offender" is included. The study notes a focus on adults, so who would qualify as a young offender?	Individuals may have been given a Young Offender sentence type, but they were all over 18 and in adult prisons during the study period. A note has been added to the bottom of the table about this.
Pg. 9: Who were the 407 people used in the analysis to assess release to the community and how are they grouped and how many? Further, what does it mean when the authors say they are analyzing data after 150 days only for the release to community analysis?	The 407 people were the individuals who had been in the open prison after 150 days from entry. We have provided details on the number of people in the PERS and non-PERS group from these 407 individuals. Due to the violation of the proportional hazard assumption, only individuals who had been in open prison for more than 150 days were included in the analysis. This has been clarified in the text within the results section when reporting the findings relating to release to the community.
Pg. 10: The following sentence is inaccurate in terms of interpretation: "PERS service users had a 48% reduced rate of being released to the community compared to the non-PERS sample" … with hazard and odds ratios, it's best to invert those ratios below 1.0 (by 1/HR) to get a more meaningful interpretation. Here, it would mean that PERS services users were 1.92 times less likely to be release to the community.	Thank you for pointing this out. We have amended the interpretation of the HR throughout to invert the ratios for greater interpretability.
Pg. 10: Who were the 507 people used in the analysis to assess return to closed custody and how are they grouped and how many? Further, what does it mean when the authors say they are analyzing data in the first 500 days? Also, improve interpretation of odds/hazard ratios below 1.0 as above.	We now clearly show in the results section before the presentation of the return to closed conditions, which of the 507 people are in the PERS and non-PERS group as well as providing clarification of what is meant by up to 500 days from entry into the open prison. The interpretation of

	the HR has been amended for greater interpretability.
For the Cox proportional hazard models, are PERS users the target category vs. the reference category of non-service users? This should be noted to avoid any confusion.	We have included the reference target category in the updated table.
the Discussion section is generally fine, but it is quite brief and limited. It would benefit from reflecting more on the current findings and drawing deeper comparisons to previous research, especially research that has been conducted on the PERS program	Thank you for suggesting this. We have added some further reflections on the current findings in relation to previous work within the discussion in relation to a lower likelihood of return to closed conditions.
It would also benefit to discuss more about what future research could/should do to assess the PERS program based on the current findings, with pointed recommendations	Thank you. Some more specific suggestions of future research studies have been provided.

VERSION 2 - REVIEW

Reviewer 1
Name Kelson, Zoe
Affiliation University of Exeter, Mathematics
Date 22-Dec-2025
COI

Thanks for the responses and revisions. The following reviewer requests are raised:

Author response:

"Consequently, a complete-case sensitivity analysis was not feasible for the matched analysis. We have clarified the nature and location of missing data and added this as a limitation in the Discussion section"

Reviewer request:

If a complete case analysis is not deemed feasible due to the amount of missing data, can an alternative sensitivity analysis on the approach taken for handling missing data please be performed to help demonstrate the robustness of the study outcomes to missing data?

Author response:

"We have also added in the discussion of limitations that an inconsistent matching ratio may have introduced confounding."

Reviewer request:

If consistent and equal matching (e.g. 1:1) is not deemed possible for sample size reasons, can weighting please be considered to adjust for any imbalance, perhaps in a sensitivity analysis?

Author response:

"Because these repeated episodes are not independent, the Cox models were clustered by individual ID to adjust for within-person correlation. We have clarified this approach within the Methods section. "

Reviewer request:

Can more detail on the clustered model methodology please be described in the article (e.g. is a partial likelihood model applied here)?

Reviewer 2

Name Stoliker, Bryce E.

Affiliation University of Saskatchewan, Centre for Forensic Behavioural Science and Justice Studies

Date 07-Jan-2026

COI

I commend the authors for the hard work put into the research study and revisions from the initial review. I believe the manuscript has improved with the revisions. A minor revision I suggest is to provide a more fulsome and detailed description of the PERS program in the Introduction section, as it is currently still significantly lacking detail for the reader to fully understand how the program operates.

VERSION 2 - AUTHOR RESPONSE

Comment	Response
Reviewer 1	
If a complete case analysis is not deemed feasible due to the amount of missing data, can an alternative sensitivity analysis on the approach taken for handling missing data please be performed to help demonstrate the robustness of the study outcomes to missing data?	Thank you for suggesting an alternative sensitivity analysis approach is taken to assess the robustness of the study outcomes in relation to the missing data outside of a complete-case analysis. As a robustness check, we tested numerous matching approaches including nearest-neighbour and optimal matching. Both methods achieved good covariate balance (all standardized mean

	differences < 0.2) and produced consistent effect estimates. Given the strong balance achieved and the stability of results across matching specifications, additional weighting-based analyses were not pursued.
If consistent and equal matching (e.g. 1:1) is not deemed possible for sample size reasons, can weighting please be considered to adjust for any imbalance, perhaps in a sensitivity analysis?	We selected our approach to matching by comparing multiple approaches. These did not include weighting to adjust for any imbalance, which would have been an interesting alternative approach.
Can more detail on the clustered model methodology please be described in the article (e.g. is a partial likelihood model applied here)?	Thank you for this comment, we agree further detail on the clustered model in the methodology is a helpful addition. Within the statistical analysis section of the methodology, we have added that the models are estimated using a partial likelihood model and that models were clustered on the individual using cluster-robust standard errors.
Reviewer 2	
A minor revision I suggest is to provide a more fulsome and detailed description of the PERS program in the Introduction section, as it is currently still significantly lacking detail for the reader to fully understand how the program operates.	Thank you for this suggestion, we agree the current description did not capture the PERS service accurately, so this has been re-written in simpler terms within the fifth paragraph of the introduction to explain the three core components of the service and how they interact.

VERSION 3 - REVIEW

Reviewer 1
Name Kelson, Zoe
Affiliation University of Exeter, Mathematics
Date 29-Jan-2026
COI

Thanks to the authors for their responses.

Author response:

"As a robustness check, we tested numerous matching approaches including nearest-neighbour and optimal matching. Both methods achieved good covariate balance (all

standardized mean differences < 0.2) and produced consistent effect estimates. Given the strong balance achieved and the stability of results across matching specifications, additional weightingbased analyses were not pursued."

Reviewer request:

Can the sensitivity analyses the authors refer to (i.e. nearest-neighbour) in their response to reviewer please be reported in the article, as this helps demonstrate the robustness of the study findings?

Author response:

"We selected our approach to matching by comparing multiple approaches. These did not include weighting to adjust for any imbalance, which would have been an interesting alternative approach."

Article text:

"Optimal matching was chosen as it minimises the average absolute distance across all matched pairs. "

and

"Matching was conducted at the episode level and therefore an individual who used PERS more than once in the study period was matched based on their covariates at the time of each episode"

and

"A four to one ratio of matching was used, identifying four non-PERS service users for each PERS service user. The matching took place prior to individuals being identified as having been in an open prison (when the data was linked to NOMIS) and therefore the final number of controls to service user ratio was less than four to one. The 4:1 ratio was therefore chosen to ensure that the control group still included a sufficient sample in the Cox Proportional hazard models."

and

"Between April 2019 and April 2022, 269 individuals were recorded as PERS users. 11 people had missing ethnicity data and were removed from the analysis, leaving 258 individuals included in the PERS user group. 258 individuals were matched to a control group of 1076 matched controls who had not used PERS. Out of the 1076 controls identified through the matching, only 358 had been in an open prison between April 2019 and April 2022, so this was the final sample size of the matched control group"

Reviewer request:

The authors have matched controls to cases prior to exclusions from both groups, which means that some controls may be included which are not matched to a PERS user (since that user was later excluded due to missing data) and some PERS users may be included with <4

(maybe 0) matched controls (due to controls being excluded based on not being in an open prison). This results in a variable matching ratio, with the possibility for some unmatched individuals in the cohort, leading to potential biases.

Can the authors please reconsider the previous reviewer request to undertake a sensitivity analysis to demonstrate the robustness of the study outcomes to the matching methdology (not just the algorithm applied, but the process followed)? Ideally matching should be conducted after applying the eligibility criteria for inclusion in the analysis.

VERSION 3 - AUTHOR RESPONSE

Reviewer comment	Response
Can the sensitivity analyses the authors refer to (i.e. nearest-neighbour) in their response to reviewer please be reported in the article, as this helps demonstrate the robustness of the study findings?	Thank you for this suggestion we have included the model and balance plots in the supplementary materials and added a line on page 6 to signpost readers towards this.
The authors have matched controls to cases prior to exclusions from both groups, which means that some controls may be included which are not matched to a PERS user (since that user was later excluded due to missing data) and some PERS users may be included with <4 (maybe 0) matched controls (due to controls being excluded based on not being in an open prison). This results in a variable matching ratio, with the possibility for some unmatched individuals in the cohort, leading to potential biases.<br><br>Can the authors please reconsider the previous reviewer request to undertake a sensitivity analysis to demonstrate the robustness of the study outcomes to the matching methodology (not just the algorithm applied, but the process followed)? Ideally matching should be conducted after applying the eligibility criteria for inclusion in the analysis.	We thank the reviewer for this important methodological point. We agree that the original matching approach, conducted prior to the application of eligibility criteria, created the potential for variable matching ratios and incomplete matched pairs. We have addressed this directly by conducting a sensitivity analysis performing propensity score matching after restricting both the PERS user group and the control group to individuals confirmed as having been in an open prison between April 2019 and April 2022.<br><br>Results of the sensitivity analysis are presented in full in Supplementary Table 3. Description of this sensitive analysis is provided on page 6/7 at the end of Study participants and setting section where the matching approach is explained.<br><br>The analysis of return to closed conditions produced directionally consistent results with the primary analysis in both unadjusted (HR = 1.23, 95% CI: 0.87 – 1.74, p = .249) and adjusted models (HR = 1.23, 95% CI: 0.86 – 1.77, p = .263). Estimates did not reach statistical significance, which is expected given the smaller sample size. The

	confidence intervals overlap considerably between analyses. For release to the community, only four events were observed in the sensitivity sample (n = 88), precluding reliable model estimation; this outcome cannot be meaningfully assessed in the sensitivity analysis and we note this as a limitation.

VERSION 4 - REVIEW

Reviewer 1

Name Kelson, Zoe

Affiliation University of Exeter, Mathematics

Date 10-Mar-2026

COI

Many thanks to the authors for responding to the latest round of reviewer comments, and reporting the required sensitivity analysis with respect to the matching methodology applied.
